# Supplementary material for: DMRforPairs: identifying Differentially Methylated Regions between unique samples using array based methylation profiles
Source: BMC Bioinformatics. 2014 May 15;15:141. doi: 10.1186/1471-2105-15-141 (PMC4046028; doi:10.1186/1471-2105-15-141)
Supplement: Additional file 2 — DMRforPairs output for the comparison of A431-MCF7 and NA17018-NA17105. Please start from the HTML files in each folder. Available via the BMC Bioinformatics website. [file 1471-2105-15-141-S2.zip › 1394847754114233_MOESM2_ESM/A431_MCF7/figures/11822.pdf]

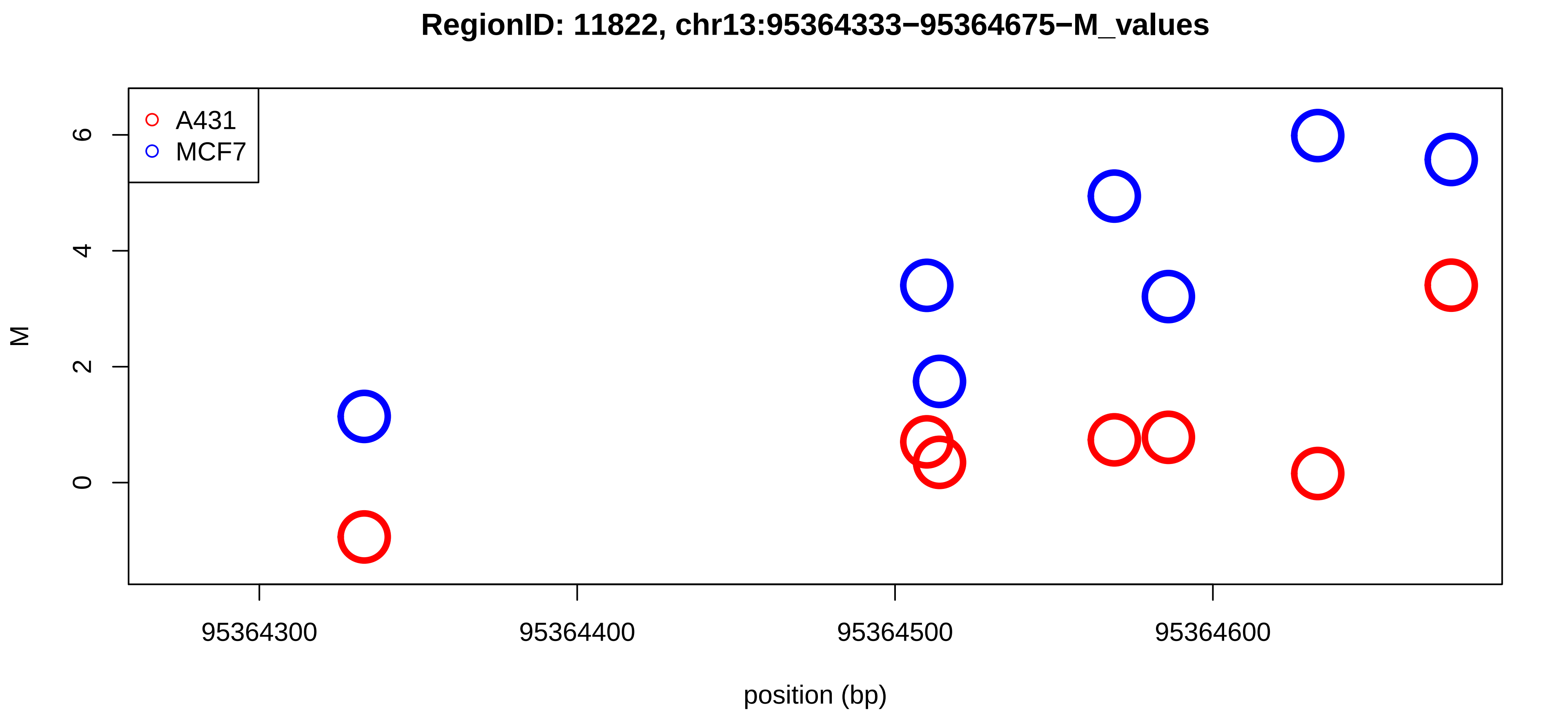

RegionID: 11822, chr13:95364333−95364675−M_values
A431
MCF7
M
position (bp)
95364300
95364400
95364500
95364600

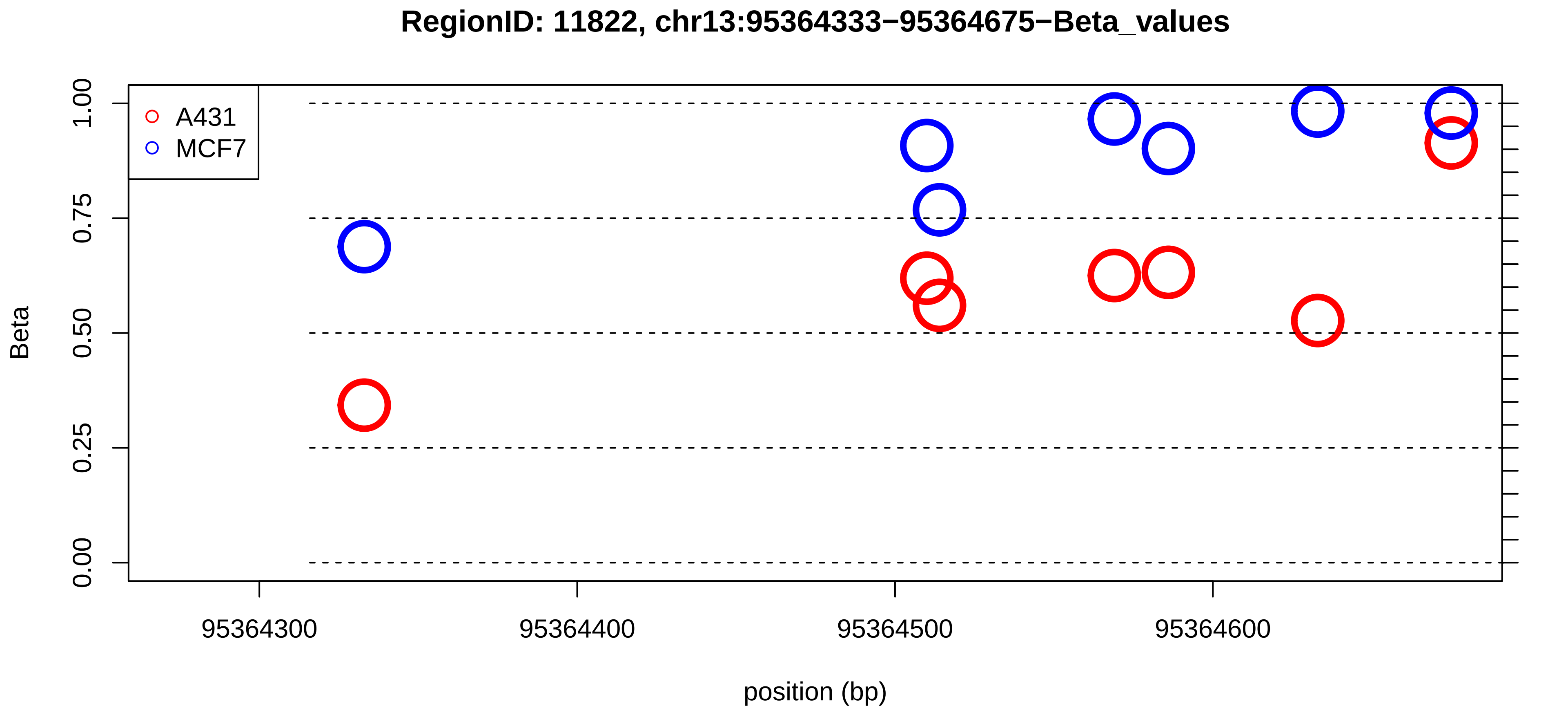

RegionID: 11822, chr13:95364333−95364675−Beta_values
A431
MCF7
Beta
position (bp)
95364300
95364400
95364500
95364600

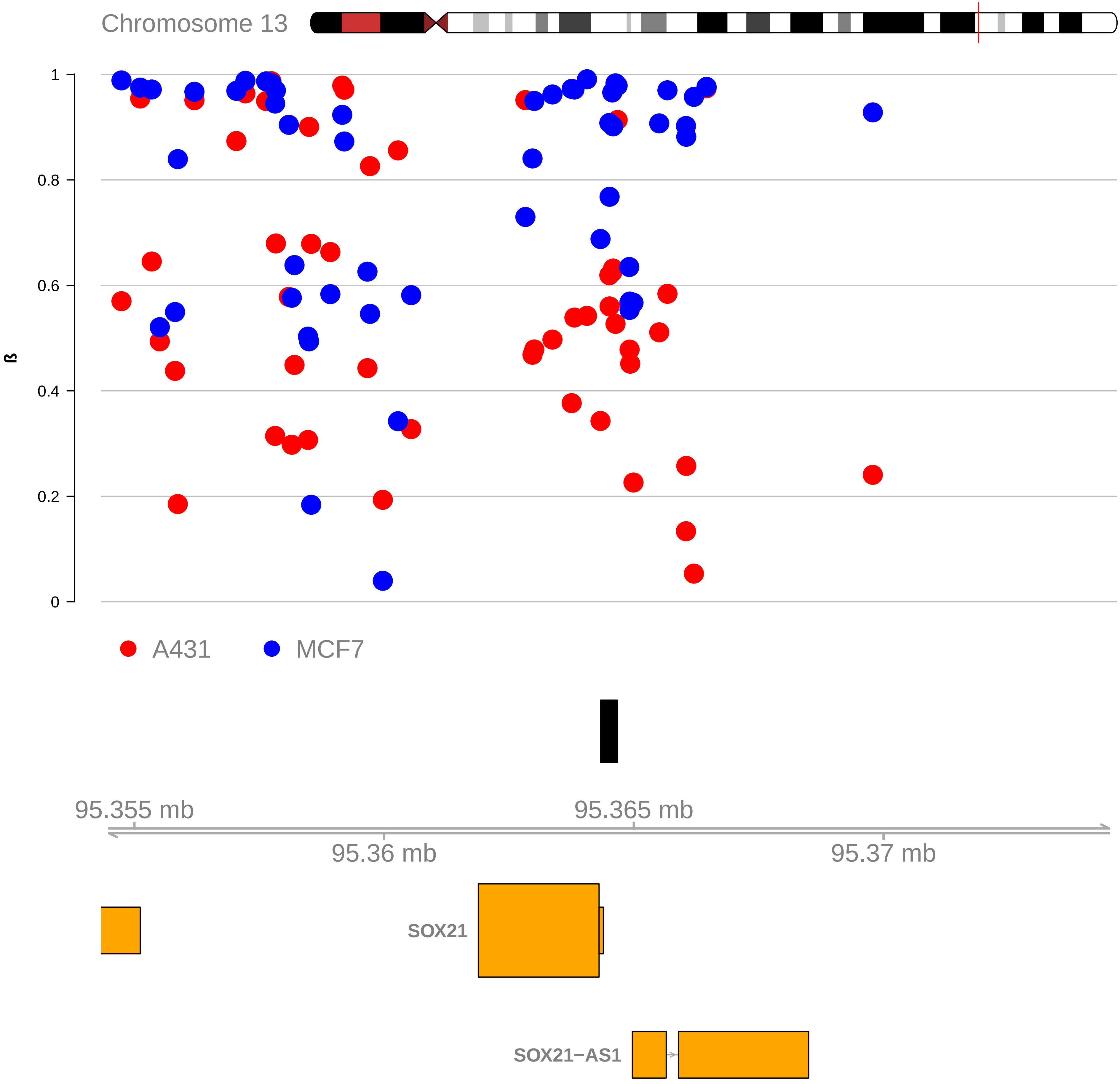

Chromosome 13
1
0.8
0.6
0.4
0.2
0
β
A431
MCF7
95.355 mb
95.365 mb
95.36 mb
95.37 mb
SOX21
SOX21−AS1
